# Human Milk-Derived Versus Bovine Protein-Based Fortifiers for Preterm Infants Fed Human Milk: A Systematic Review and Meta-Analysis

**DOI:** 10.3390/nu18193288

**Published:** 2026-10-07

**Authors:** Luling Lin, Nicholas D. Embleton, Chris H. P. Van Den Akker, Julie Brown, Lilia Delgado Paramo, Barbara E. Cormack

**Affiliations:** 1 Liggins Institute, University of Auckland, Auckland 1023, New Zealand; 2 Population Health Sciences Institute, Newcastle University, Newcastle upon Tyne NE1 4LP, UK; 3 Department of Pediatrics-Neonatology, Emma Children’s Hospital, Amsterdam UMC, University of Amsterdam, 1005 AZ Amsterdam, The Netherlands; 4 Amsterdam Reproduction and Development Research Institute, 1005 AZ Amsterdam, The Netherlands; 5 Covidence, Future Evidence Foundation, Melbourne 3000, Australia; 6 Starship Child Health, Auckland City Hospital, Auckland 1023, New Zealand

**Keywords:** preterm infant, necrotising enterocolitis, breast milk fortifier, multinutrient fortification, donor human milk, systematic review

## Abstract

**Background:** Human milk-derived fortifiers (HumBMF) are an alternative to bovine protein-based fortifiers (BovBMF) for preterm infants, but their comparative effectiveness and safety remain uncertain. We evaluated the effects of HumBMF versus BovBMF in preterm or very low birth weight infants. **Methods:** Randomised controlled trials (RCTs) comparing use of HumBMF versus BovBMF to fortify human milk were identified through four databases and three trial registries (to 11 May 2026). At least two reviewers independently screened studies, extracted data, and assessed risk of bias. Fixed-effect meta-analyses and GRADE assessments were performed. Prespecified analyses separated trials comparing fortifier type alone, in which any mother’s own milk (MOM) shortfall was supplemented with donor human milk (DHM) in both arms; and dual-intervention trials, in which MOM shortfall was also randomised to DHM in the HumBMF arm or preterm formula in the BovBMF arm. **Results:** Nine RCTs were included. In trials comparing fortifier type alone, there was no evidence that HumBMF reduced mortality (RR 0.60 (95% CI 0.29–1.23), low-certainty) or necrotising enterocolitis (NEC; RR 0.83 (0.39–1.78), very low-certainty). In dual-intervention trials, where MOM shortfall was supplemented with DHM rather than formula, NEC was lower (RR 0.45 (0.20–0.99), moderate-certainty). **Conclusions:** Current evidence does not demonstrate clinical advantages of HumBMF over BovBMF when added to the same human milk diet. Reductions in NEC occurred only where DHM also replaced preterm formula and cannot be attributed to fortifier type alone. Future research should distinguish the independent effects of fortifier composition from those of an exclusive human milk feeding strategy.

## 1. Introduction

Mother’s own milk (MOM) is widely recognised as the optimal source of enteral nutrition for very preterm infants due to its association with improved neurodevelopmental outcomes and reduced risks of infection and necrotising enterocolitis (NEC) [1,2,3,4]. However, despite its benefits, MOM alone may not meet the high nutrient requirements of extremely and very preterm infants, particularly for protein, energy, calcium and phosphate [1,5]. Therefore, multinutrient breast milk fortifier (BMF) is routinely added to expressed human milk (HM) to support adequate growth, bone mineralisation, and neurodevelopment [6].

When MOM is insufficient or unavailable, donor human milk (DHM) is increasingly preferred over preterm formula in many neonatal intensive care units (NICUs), although availability and practices vary between settings [7,8]. This reflects growing consensus that HM, even when pasteurised, confers bioactive benefits. This is particularly relevant for the most vulnerable infants, including those at highest risk of complications such as NEC. Within this context, interest has grown in the use of human milk-derived fortifiers (HumBMF) as an alternative to bovine protein-based fortifiers (BovBMF), with the aim of providing a so-called exclusively human milk diet.

The rationale for preferential use of HumBMF is based on extensive observational evidence linking exposure to bovine protein-based infant formula with an increased risk of NEC, although causation cannot be determined and the increased risks may simply reflect lower exposure to HM. NEC is associated with substantial mortality, long-term morbidity, and health system costs [9]. Randomised controlled trials (RCTs) and meta-analyses suggest that DHM reduces the risk of NEC, but not mortality, although many uncertainties remain [4].

HumBMF are commercially available in several countries, particularly in North America, but their use is constrained by high costs, limited availability and variability in regulatory and ethical frameworks governing the use of human tissue-derived products, including breast milk [10]. In addition, there are important considerations regarding how HM is sourced, processed and transformed into commercial products. HumBMF are typically manufactured from pooled human milk that undergoes extensive screening, processing, concentration, and pasteurisation, with minerals added to achieve the desired nutritional composition [11]. The terminology surrounding milk sourcing also warrants consideration, as some commercial programmes provide financial compensation to women supplying HM, differing from the voluntary, non-remunerated donation model traditionally associated with DHM banking.

These practices also raise broader societal and cultural considerations, including transparency around which women provide milk and their motivations, whether their own children may miss out on breast milk, the commodification of human milk, equity of access, and the governance of commercial entities involved in the procurement and manufacture of these products [12].

Many currently available commercial HumBMF are produced as liquid formulations, and when used clinically, they displace a proportion (typically 30–50%) of milk volume and thereby decrease overall exposure to MOM. However, several observational studies have reported associations between exclusive human milk feeding strategies incorporating HumBMF and lower rates of NEC and other neonatal morbidities [13,14]. Conversely, observational studies have also reported metabolic abnormalities following the introduction of HumBMF in extremely preterm infants, including hypoglycaemia, hyperphosphataemia, and hypercalcaemia compared with BovBMF, although causality remains uncertain [15]. Together, these scientific and societal issues contribute to ongoing uncertainty about the optimal role of HM products in neonatal care, especially as costs, healthcare funding, access, and regulatory oversight vary globally.

Given the clinical importance of NEC, the high financial costs and other uncertainties associated with HumBMF, our aim was to systematically evaluate the controlled trial evidence comparing HumBMF with BovBMF in preterm infants. We aimed to include all studies in which fortification was randomised to either HumBMF or BovBMF, which may include studies where the milk used to make up any shortfall in MOM may also have been randomised (e.g., to DHM or formula).

## 2. Methods

This review was conducted according to the Cochrane Handbook for Systematic Reviews of Interventions [16] and reported in line with the Preferred Reporting Items for Systematic Reviews and Meta-Analyses (PRISMA) checklist [17] (Supplemental File S1) and prospectively registered with PROSPERO (registration number CRD42024600039).

### 2.1. Search Strategy and Selection Criteria

The following electronic databases were searched from inception to 11 May 2026: MEDLINE (Ovid), Embase (Ovid), CINAHL Ultimate, and the Cochrane Central Register of Controlled Trials (CENTRAL). Registered trials were also searched for in: Clinical Trials (www.ClinicalTrials.gov, accessed on 11 May 2026), WHO ICTRP Search Portal (https://apps.who.int/trialsearch/, accessed on 11 May 2026), and Australia New Zealand Clinical Trial Registry (https://www.anzctr.org.au/, accessed on 11 May 2026). There were no language and publication date restrictions. Conference abstracts were included if they provided usable summary data. Reference lists of included studies were also screened. See Supplementary File S2 for the full search strategy.

We included randomised and quasi-randomised controlled trials comparing HumBMF with BovBMF for fortification of HM in preterm infants (<37 weeks’ gestation). Trials in which MOM shortfalls were supplemented with DHM in the HumBMF arm but with preterm formula in the BovBMF arm were analysed separately as a prespecified subgroup, because these trials evaluate a combined feeding strategy rather than the independent effect of fortifier type. The primary outcome was mortality before discharge from neonatal care. Secondary outcomes included NEC (modified Bell’s stage ≥ 2), NEC requiring surgery, retinopathy of prematurity (ROP; confirmed and requiring treatment), confirmed or suspected sepsis, bronchopulmonary dysplasia (BPD; oxygen or ventilatory support at 36 weeks’ corrected age, or defined by authors), metabolic bone disease of prematurity, neurodevelopmental outcomes, feeding intolerance (as defined by authors), time to full enteral feeds, growth (weight, length, or head circumference gain) and length of hospital stay.

### 2.2. Data Collection and Analysis

Two of four review authors (BC, CA, NE, and LL) independently screened titles and abstracts of identified records, then assessed potentially eligible full-text articles for inclusion using Covidence [18]. The same review authors then independently extracted data into a pre-specified data extraction form. The extracted data included the authors, study setting, study methodology, ethics approval, conflicts of interest, funding sources, information for the assessment of the risk of bias, participant characteristics, intervention and control details, and outcome data. Discrepancies at each step were resolved by discussion between the reviewers.

The review authors independently assessed risk of bias using the Cochrane Risk of Bias 2 (ROB-2) tool [19], covering the domains of randomisation, deviations from intended interventions, missing outcome data, outcome measurement, reporting, and overall bias. Judgements were categorised as low risk, some concerns, or high risk. Disagreements were resolved through discussion or with a third reviewer.

Review authors who were investigators or co-authors of an included study were not involved in eligibility decisions, data extraction, or risk-of-bias assessment for that study.

### 2.3. Data Synthesis

RevMan 5.4 [20] was used to synthesise data. For dichotomous outcomes, we calculated relative risks (RRs) with 95% confidence intervals (CIs). For continuous outcomes, we calculated mean differences (MDs) with 95% CIs. A *p*-value < 0.05 was considered statistically significant. We assessed whether studies were sufficiently comparable for meta-analysis. Studies were stratified according to whether the control group received DHM or preterm formula when there was any MOM shortfall, as these comparator feeding strategies were considered clinically distinct. Overall pooled estimates across these strata were not calculated. Although the registered protocol specified random-effects models, within strata, fixed-effect models were used because studies were sufficiently similar in population and overall dietary comparison to support estimation of a common underlying effect, and the small number of trials within each stratum precluded reliable estimation of between-study variance for random-effects models. Heterogeneity was quantified using the *I*^2^ and χ^2^ statistics; where heterogeneity was substantial, estimates were interpreted accordingly. Post hoc sensitivity analyses using random-effects models were conducted for outcomes included in the GRADE Summary of Findings tables where at least two trials contributed data, to assess the robustness of findings to the choice of meta-analysis model. Publication bias assessment using funnel plots was planned when at least 10 studies were available.

### 2.4. Certainty of Evidence

We aimed to determine the certainty of evidence for the primary outcome and for prespecified key secondary outcomes of clinical importance and where sufficient data was available: mortality before discharge, NEC (modified Bell’s stage ≥ 2), NEC requiring surgery, ROP, sepsis, BPD, neurodevelopmental impairment and feeding intolerance using the Grading of Recommendations Assessment, Development and Evaluation (GRADE) approach [21]. Two “Summary of Findings” tables were compiled using the GRADEpro Guideline Development Tool (GDT) [22], one is for the trials where MOM or DHM was maintained as the baseline diet in both groups, and the other is for the trials where the control group (BovBMF) received preterm formula when there was any MOM shortfall.

### 2.5. Subgroup Analyses

We aimed to perform subgroup analyses by gestational age category (extremely preterm [<28 weeks], very preterm [28–32 weeks], and moderate-to-late preterm [32–37 weeks]); however, insufficient data were available to support these analyses.

## 3. Results

### 3.1. Characteristics of Included Studies

**Figure 1:**
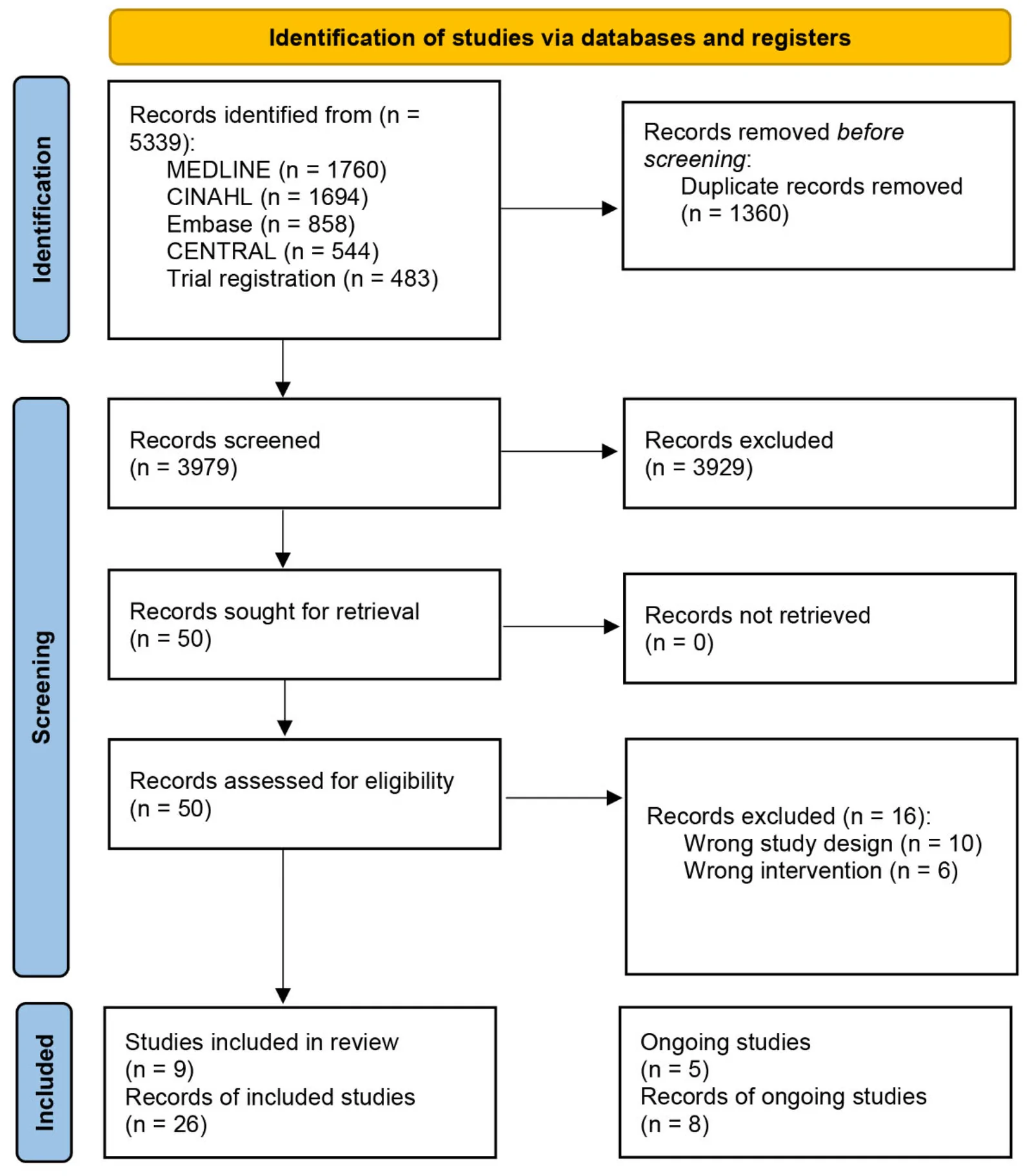
Flow chart of selection of eligible studies.

In total, the search identified 5339 records. After removing duplicates, we screened 3979 titles and abstracts and assessed 50 full texts. Nine studies (26 records) met the inclusion criteria (Figure 1); an additional five ongoing studies (eight records) were identified and are listed with their trial registration numbers in Supplementary File S3. Included studies were published between 1993 and 2026 and conducted in the UK (*n* = 2), Sweden (*n* = 2), Brazil (*n* = 1), Canada (*n* = 1), Japan (*n* = 1), and as multicentre trials across Italy and Germany (*n* = 1) and the USA and Austria (*n* = 1). All included infants were extremely or very preterm, or had extremely or very low birth weight. In six studies [23,24,25,26,27,28], MOM or DHM was maintained as the baseline diet in both groups, whereas in the remaining three studies the control group (BovBMF) received preterm formula when there was MOM shortfall [29,30,31] (Table 1).

### 3.2. Risk of Bias

**Figure 2:**
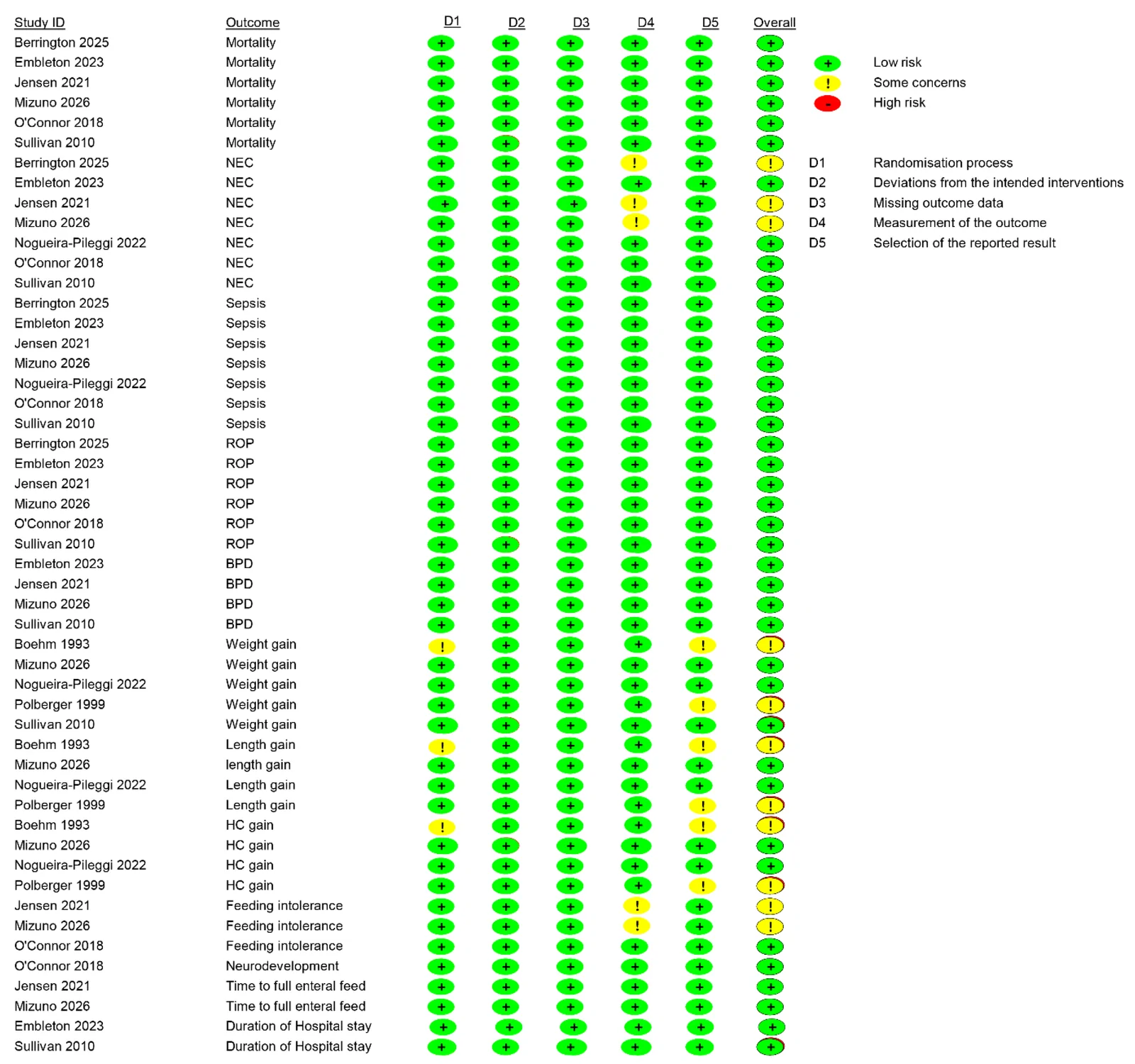
Risk of bias assessment using ROB2. (Abbreviations: NEC, Necrotising enterocolitis; ROP, Retinopathy of prematurity; BPD, Bronchopulmonary dysplasia; HC, Head circumference).

Risk of bias was assessed at the outcome level using the RoB2 tool [19] (Figure 2). Most outcomes were judged to be at low risk of bias across all domains. Although several trials were open label after randomisation, major clinical outcomes, including mortality, ROP, BPD, growth outcomes, time to full enteral feeds, and duration of hospital stay, were considered unlikely to be influenced by knowledge of treatment allocation because they were objective outcomes or were assessed using predefined criteria. NEC outcomes reported by Berrington et al. [23], Jensen et al. [25], and Mizuno et al. [30] were judged to have some concerns because the trials were open label, blinding of NEC outcome assessment was not reported, and NEC diagnosis and staging involve interpretation of clinical and radiographic findings that could potentially be influenced by knowledge of treatment allocation [32]. Some concerns were identified for growth outcomes reported by Boehm et al. [24] due to insufficient information regarding the randomisation process and selection of the reported results. Growth outcomes reported by Polberger et al. [28] were also judged to have some concerns because no prespecified protocol or statistical analysis plan was available to assess the risk of selective reporting. Feeding intolerance outcomes reported by Jensen et al. [25] and Mizuno et al. [30] were judged to have some concerns because outcome assessment may have been influenced by the lack of blinding of clinical staff. No outcome was judged to be at high risk of bias.

### 3.3. Primary Outcome—Mortality

**Figure 3:**
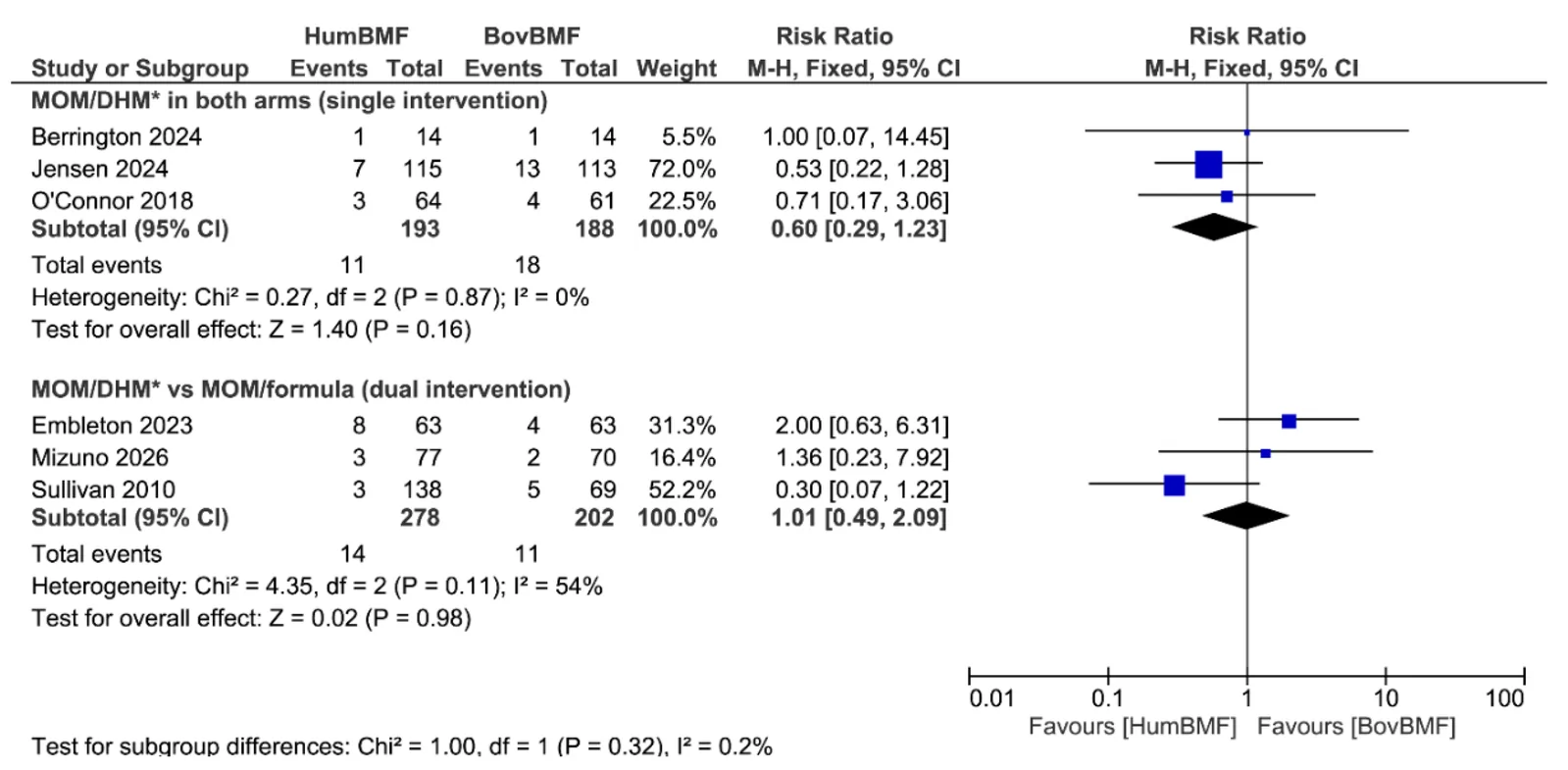
Effect of human milk-derived fortifier versus bovine protein-derived fortifier on mortality before hospital discharge. (Abbreviations: HumBMF, human milk-derived fortifier; BovBMF, bovine protein-derived fortifier; MOM, mother’s own milk; DHM, donor human milk. * DHM may be ‘commercial DHM’).

In trials where MOM or DHM was maintained as the baseline diet in both arms, HumBMF may reduce mortality, but the confidence interval is consistent with both a substantial reduction and an increase in mortality (3 RCTs, 381 infants, RR 0.60 95% CI (0.29, 1.23), *p* = 0.16, *I*^2^ = 0%, low certainty). In trials where MOM shortfall was randomised to DHM in the HumBMF arm or formula in the BovBMF arm (hereafter dual-intervention trials), the evidence is very uncertain about the effect of HumBMF on mortality (3 RCTs, 480 infants, RR 1.01 95% CI (0.49, 2.09), *p* = 0.98, *I*^2^ = 54%, very low certainty). There was no evidence that the effect of HumBMF differed according to whether HM (either MOM or DHM) was maintained as the baseline diet in both arms, or whether formula was used to supplement MOM shortfall in the control group (interaction *p* = 0.32) (Figure 3).

### 3.4. Secondary Outcomes

#### 3.4.1. Clinical Outcomes

**Figure 4:**
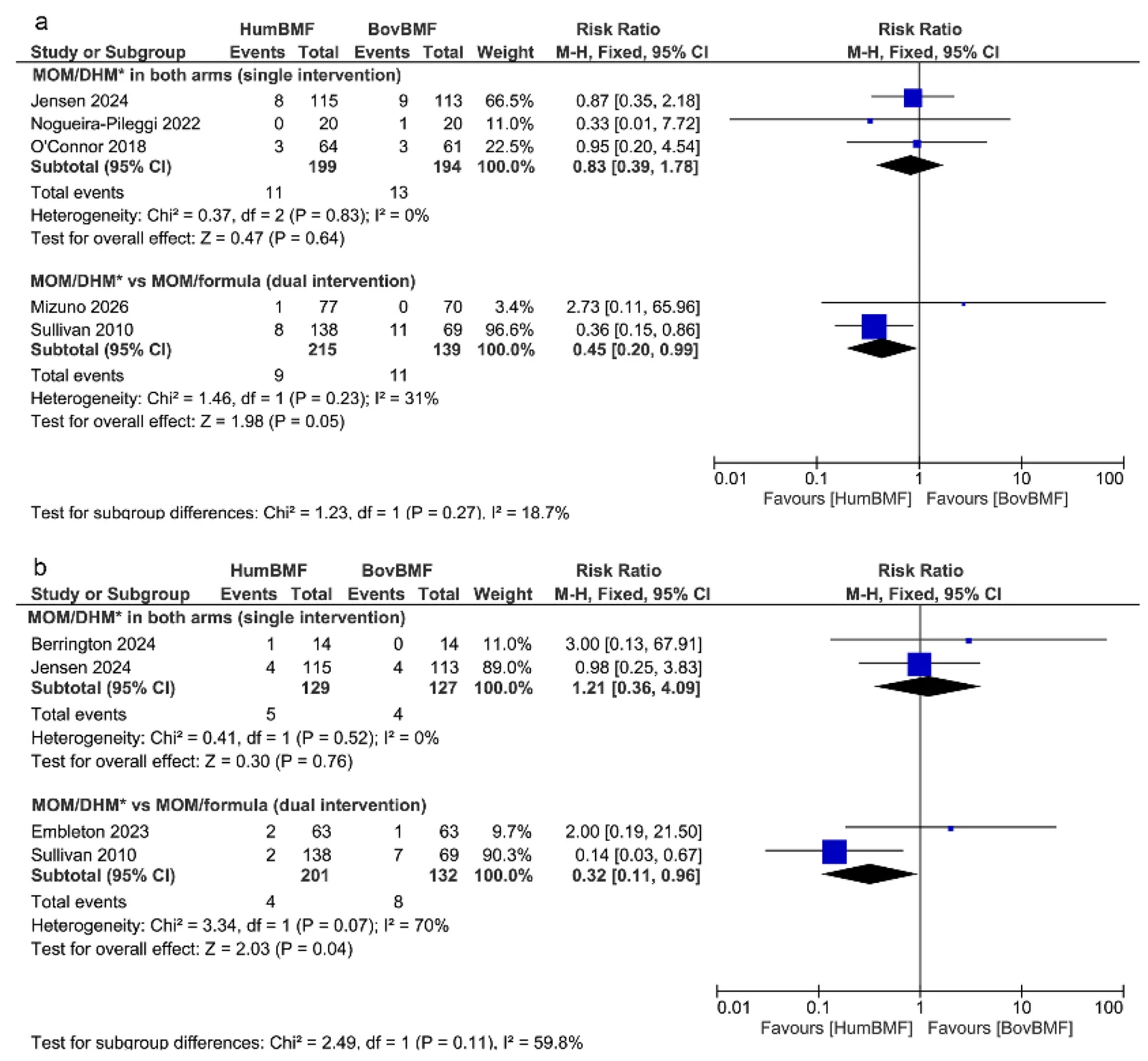

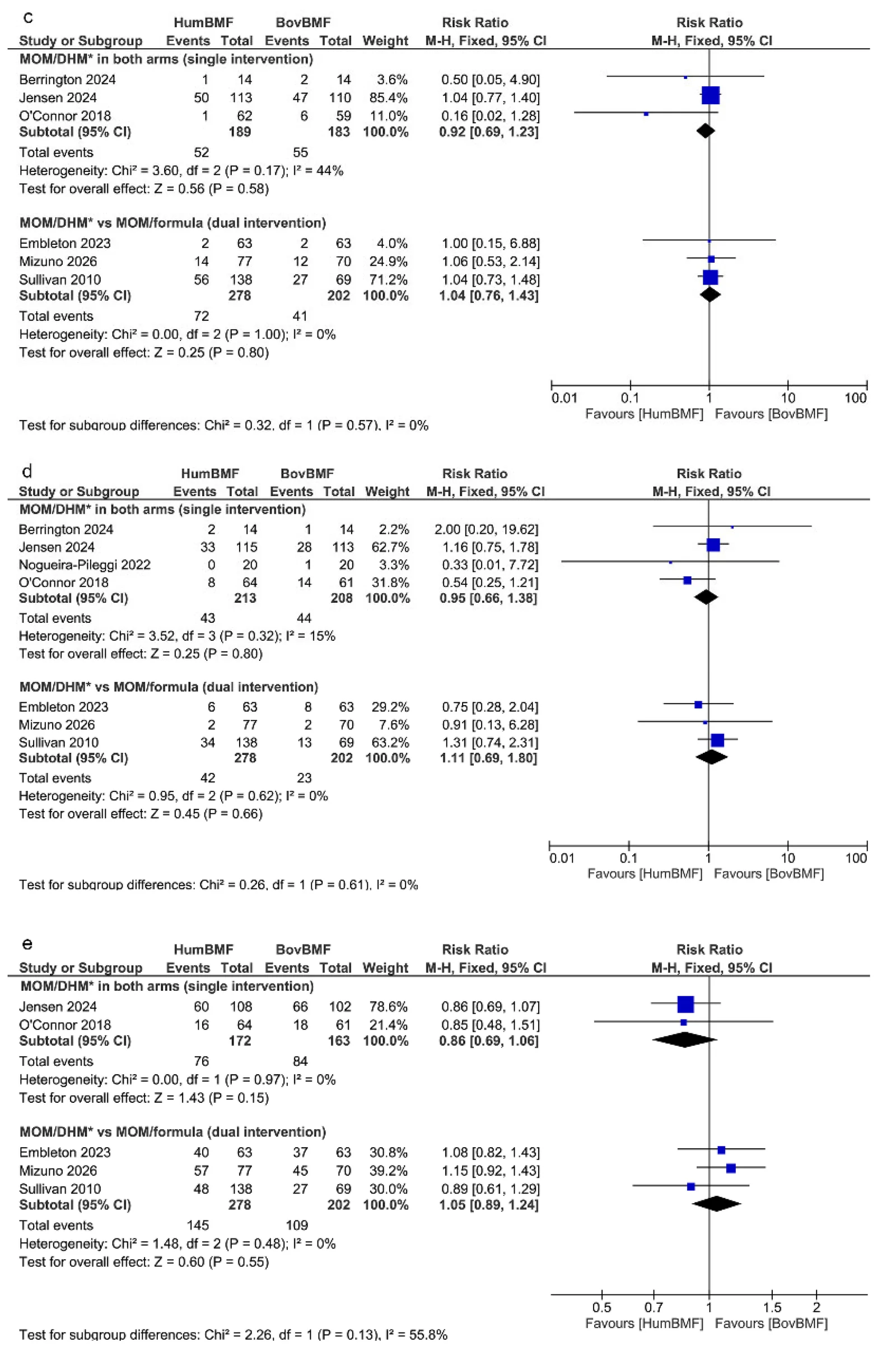
Effect of human milk-derived fortifier versus bovine protein-derived fortifier on clinical outcomes. (**a**) Necrotising enterocolitis modified Bell’s stage ≥ 2; (**b**) Necrotising enterocolitis requiring surgery; (**c**) Retinopathy of prematurity; (**d**) sepsis; (**e**) Bronchopulmonary dysplasia. (Abbreviations: HumBMF, human milk-derived fortifier; BovBMF, bovine protein-derived fortifier; MOM, mothers’ own milk; DHM, donor human milk. * DHM may be commercial ‘DHM’).

In trials where MOM or DHM was maintained as the baseline diet in both arms, the evidence is very uncertain about the effect of HumBMF on NEC (modified Bell’s stage ≥2, 3 RCTs, 393 infants, RR 0.83 (0.39, 1.78), *p* = 0.64, *I*^2^ = 0%, very low certainty). In dual-intervention trials, this combined intervention (HM and HumBMF) probably reduces NEC (2 RCTs, 354 infants, RR 0.45 (0.20, 0.99), *p* = 0.05, *I*^2^ = 31%, moderate certainty). However, the independent effect of HumBMF cannot be separated from the effect of milk type. There was no evidence that the effect of HumBMF differed according to whether HM (either MOM or DHM) was maintained as the baseline diet in both arms, or whether formula was used to supplement MOM shortfall in the control group (interaction *p* = 0.27) (Figure 4a).

In trials where MOM or DHM was maintained as the baseline diet in both arms, the evidence is very uncertain about the effect of HumBMF on NEC requiring surgery (2 RCTs, 256 infants, RR 1.21 (0.36, 4.09), *p* = 0.76, *I*^2^ = 0%, very low certainty). In dual-intervention trials, this combined intervention (HM and HumBMF) may reduce NEC requiring surgery (2 RCTs, 333 infants, RR 0.32 (0.11, 0.96), *p* = 0.04, *I*^2^ = 70%, low certainty). There was no evidence that the effect of HumBMF differed according to whether HM (either MOM or DHM) was maintained as the baseline diet in both arms, or whether formula was used to supplement insufficient MOM in the control group (interaction *p* = 0.11) (Figure 4b).

HumBMF probably results in little to no effect in ROP in trials where MOM or DHM was maintained as the baseline diet in both arms (3 RCTs, 372 infants, RR 0.92 (0.69, 1.23), *p* = 0.58, *I*^2^ = 44%, moderate certainty) or in dual-intervention trials (3 RCTs, 480 infants, RR 1.04 (0.76, 1.43), *p* = 0.80, *I*^2^ = 0%, moderate certainty). There was no evidence that the effect of HumBMF differed according to whether HM (either MOM or DHM) was maintained as the baseline diet in both arms, or whether formula was used to supplement insufficient MOM in the control group (interaction *p* = 0.57) (Figure 4c).

HumBMF probably result in little to no effect in sepsis in trials where MOM or DHM was maintained as the baseline diet in both arms (4 RCTs, 421 infants, RR 0.95 (0.66, 1.38), *p* = 0.80, *I*^2^ = 15%, moderate certainty) or in dual-intervention trials (3 RCTs, 480 infants, RR 1.11 (0.69, 1.80), *p* = 0.66, *I*^2^ = 0%, moderate certainty). There was no evidence that the effect of HumBMF differed according to whether HM (either MOM or DHM) was maintained as the baseline diet in both arms, or whether formula was used to supplement insufficient MOM in the control group (interaction *p* = 0.61) (Figure 4d).

HumBMF probably results in little to no effect on BPD in trials where MOM or DHM was maintained as the baseline diet in both arms (2 RCTs, 335 infants, RR 0.86 (0.69, 1.06), *p* = 0.15, *I*^2^ = 0%, moderate certainty) or in dual-intervention trials (3 RCTs, 480 infants, RR 1.05 (0.89, 1.24), *p* = 0.55, *I*^2^ = 0%, high certainty). There was no evidence that the effect of HumBMF differed according to whether HM (either MOM or DHM) was maintained as the baseline diet in both arms, or whether formula was used to supplement insufficient MOM in the control group (interaction *p* = 0.13) (Figure 4e).

No studies reported data for metabolic bone disease of prematurity.

#### 3.4.2. Neurodevelopmental Outcomes

Only one study [27], in which MOM or DHM was maintained as the baseline diet in both arms, reported neurodevelopmental outcomes at 18 months’ follow-up [33], and the certainty of the evidence was low. HumBMF may result in little to no effect in Bayley III cognitive score < 85 (116 infants; RR 1.09 (0.56, 2.12); *p* = 0.80), language score < 85 (113 infants; RR 0.89 (0.56, 1.43); *p* = 0.64), or motor score < 85 (115 infants; RR 1.03 (0.52, 2.04); *p* = 0.93). Similarly, HumBMF may result in little to no effect in more severe impairment, including Bayley III cognitive score < 70 (116 infants; RR 0.87 (0.37, 2.04); *p* = 0.75), language score < 70 (113 infants; RR 0.93 (0.45, 1.93), *p* = 0.85), or motor score < 70 (115 infants; RR 0.71 (0.30, 1.66), *p* = 0.43).

#### 3.4.3. Feeding Outcomes

**Figure 5:**
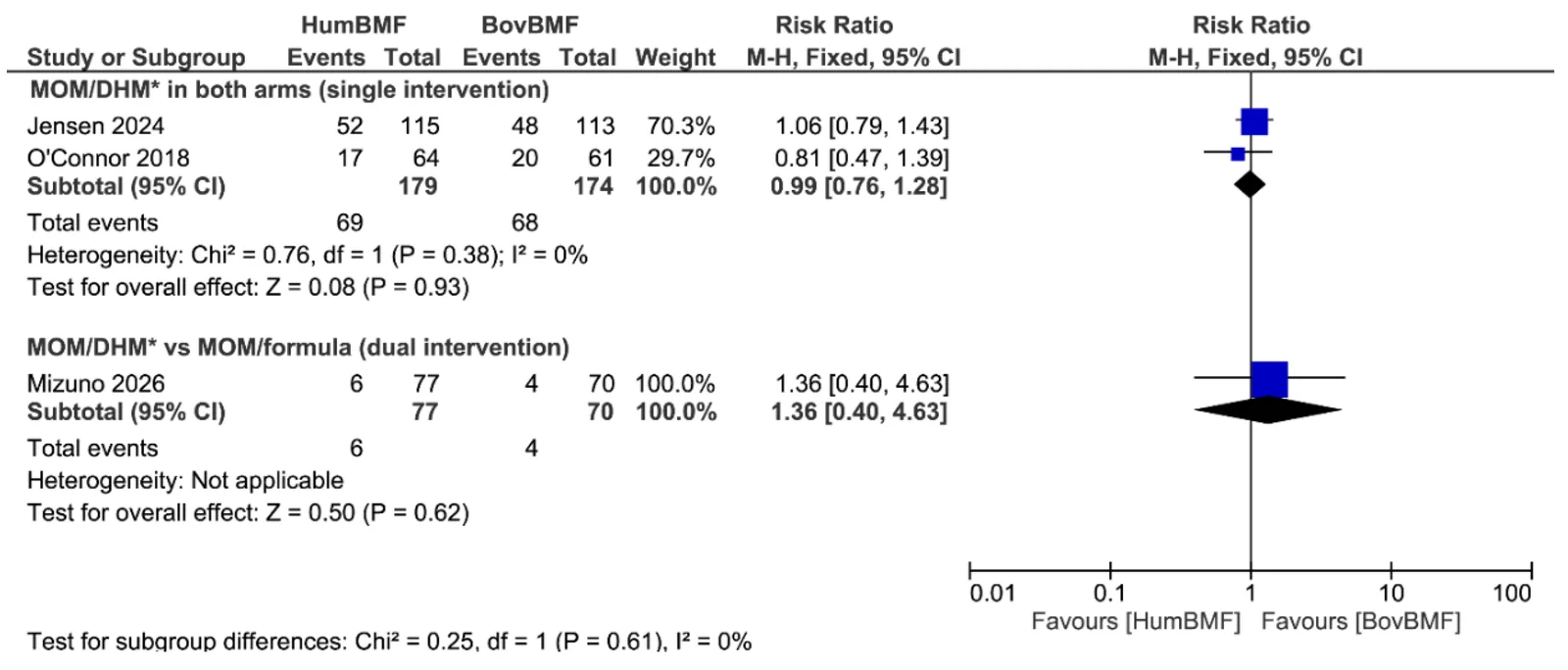
Effect of human milk-derived fortifier versus bovine protein-derived fortifier on feeding intolerance. (Abbreviations: HumBMF, human milk-derived fortifier; BovBMF, bovine protein-derived fortifier; MOM, mother’s own milk; DHM, donor human milk. * DHM may be ‘commercial DHM’).

HumBMF may result in little to no effect on feeding intolerance in trials where MOM or DHM was maintained as the baseline diet in both arms (2 RCTs, 353 infants, RR 0.99 (0.76, 1.28), *p* = 0.93, *I*^2^ = 0%, low certainty) (Figure 5). Both studies (Jensen 2024 and O’Connor 2018) [25,27] defined feeding intolerance as feeds withheld for ≥12 h or reduced by >50% in volume.

In a dual-intervention trial [30], the evidence is very uncertain about the effect of HumBMF on feeding intolerance (1 RCT, 147 infants, RR 1.36 (0.40, 4.63), *p* = 0.62, very low certainty, Figure 4). In addition, the study did not provide a definition of feeding intolerance.

There was no evidence that the effect of HumBMF differed according to whether HM (either MOM or DHM) was maintained as the baseline diet in both arms, or whether formula was used to supplement insufficient MOM in the control group (interaction *p* = 0.61).

In Jensen 2024 [25], where MOM or DHM was maintained as the baseline diet in both arms, HumBMF may result in little to no effect on the time to achieve full enteral feeds (150 mL/kg/day) (217 infants, MD 1.0 day (−0.27, 2.27); *p* = 0.12). In contrast, Mizuno 2026 [30], where, in addition to randomisation of type of fortifier, insufficient MOM was supplemented with DHM in the intervention arm or preterm formula in the control arm, infants receiving the dual intervention reached full enteral feeds (160 mL/kg/day) earlier than controls (67 infants, MD −5.90 days (−11.41, −0.39); *p* = 0.04). As this trial evaluated both fortifier type and supplemental milk, the earlier achievement of full enteral feeds cannot be attributed to HumBMF alone. A significant interaction between trials where HM (either MOM or DHM) was maintained as the baseline diet in both arms, or whether formula was used to supplement insufficient MOM in the control group (interaction *p* = 0.02) was observed.

#### 3.4.4. Growth Outcomes

One study [26], where HM (either MOM or DHM) was maintained as the baseline diet in both arms, reported growth outcomes at day 21 (40 infants). HumBMF may result in little to no difference in weight (MD −113.20 g (−276.33, 49.93), *p* = 0.17), length (MD −0.91 cm (−2.17, 0.35), *p* = 0.16), or head circumference (MD 0.15 cm, (−1.20, 1.50), *p* = 0.83).

**Figure 6:**
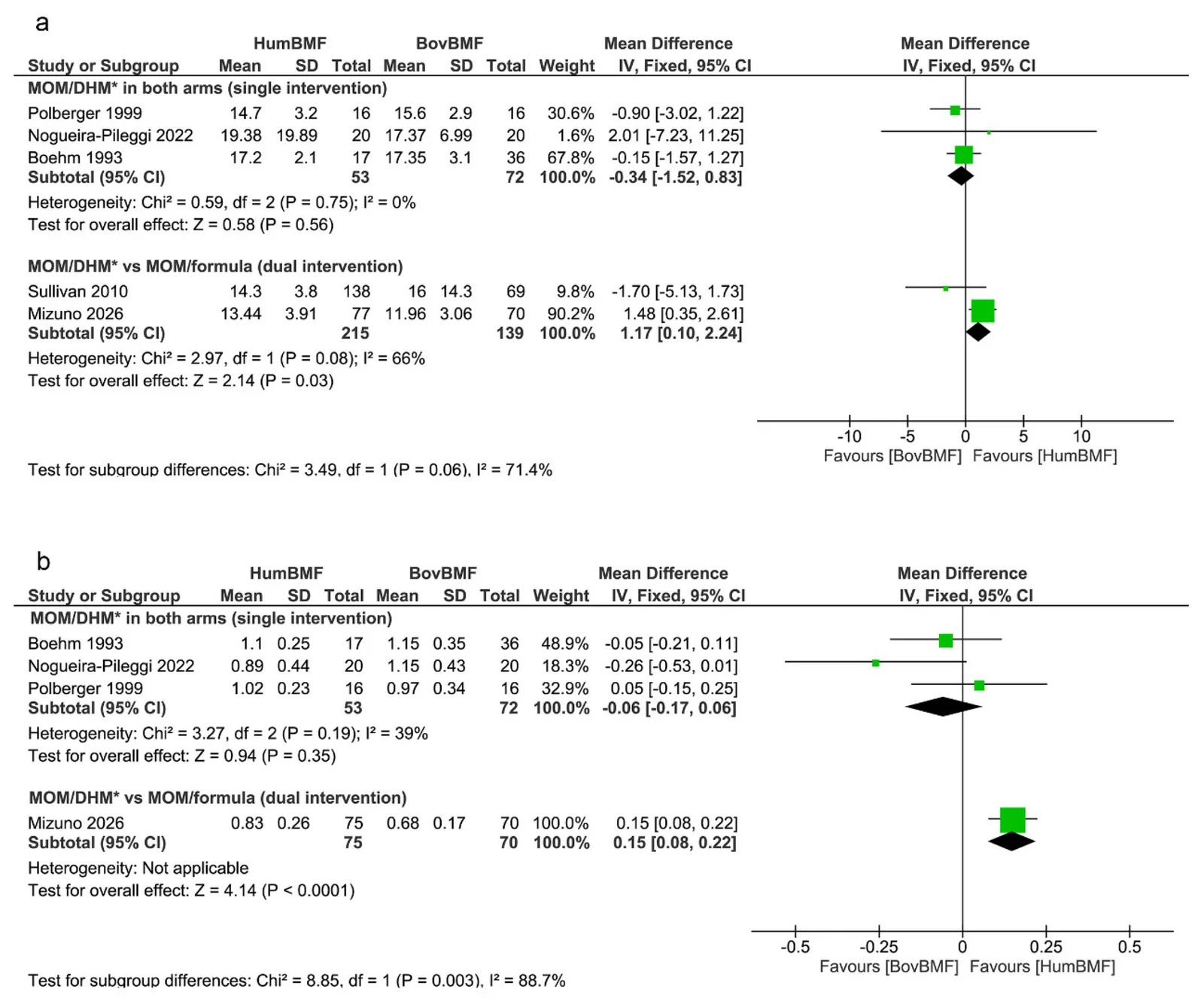

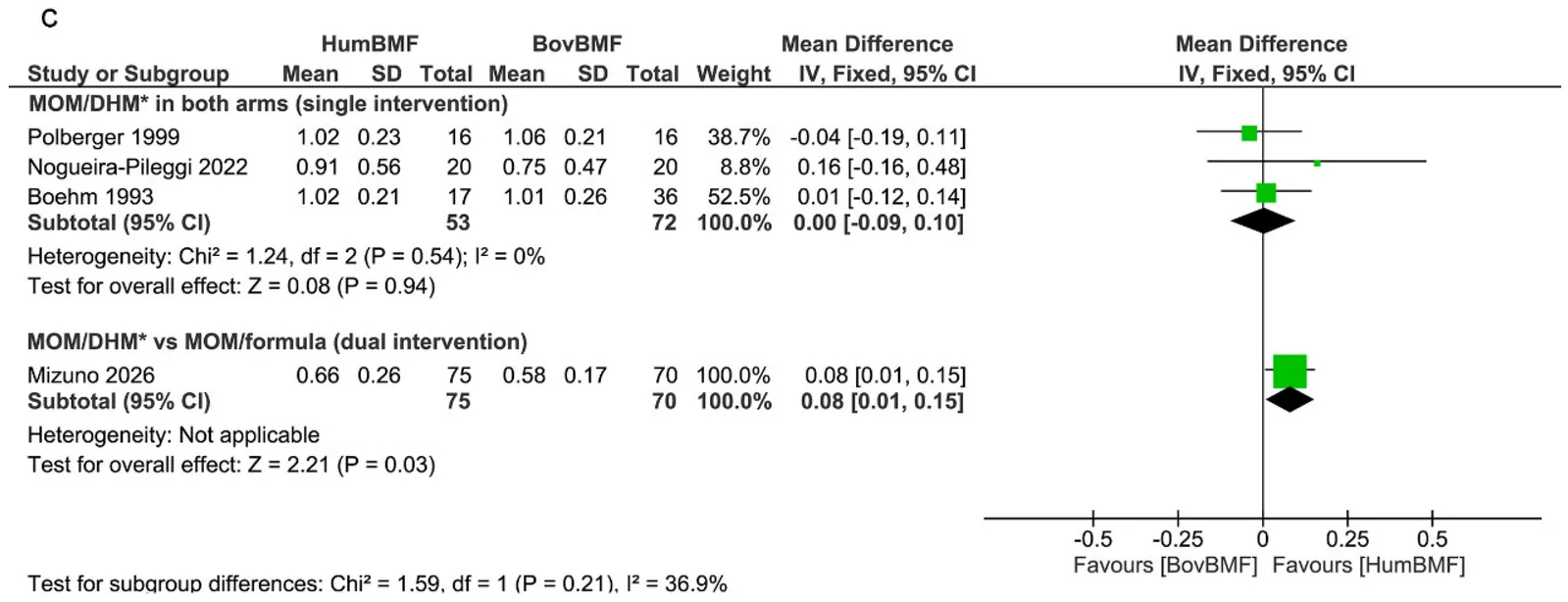
Effect of human milk-derived fortifier versus bovine protein-based fortifier on growth outcomes. (**a**) weight gain velocity (g/kg/day); (**b**) mean length gain velocity (cm/week); (**c**) head circumference gain velocity (cm/week) (Abbreviations: HumBMF, human milk-derived fortifier; BovBMF, bovine protein-based fortifier; MOM, mother’s own milk; DHM, donor human milk. * DHM may be ‘commercial DHM’).

In trials where MOM or DHM was maintained as the baseline diet in both arms, HumBMF may result in little to no effect on weight gain (3 RCTs, 125 infants, MD −0.34 g/kg/day (−1.52, 0.83), *p* = 0.56, *I*^2^ = 0%). However, in dual-intervention trials, this combined intervention (HM and HumBMF) may increase weight gain (2 RCTs, 354 infants, MD 1.17 g/kg/day (0.10, 2.24), *p* = 0.03, *I*^2^ = 66%). There was no evidence that the effect of HumBMF differed according to whether HM (either MOM or DHM) was maintained as the baseline diet in both arms, or whether formula was used to supplement insufficient MOM in the control group (interaction *p* = 0.06) (Figure 6a).

In trials where MOM or DHM was maintained as the baseline diet in both arms, HumBMF may result in little to no effect on length gain (3 RCTs, 125 infants, MD −0.06 cm/week (−0.17, 0.06), *p* = 0.35, *I*^2^ = 39%). However, in dual-intervention trials, this combined intervention (HM and HumBMF) may increase length gain (1 RCT, 145 infants, MD 0.15 cm/week (0.08, 0.22), *p* < 0.0001). There was a significant interaction according to whether HM (either MOM or DHM) was maintained as the baseline diet in both arms, or whether formula was used to supplement insufficient MOM in the control group (interaction *p* = 0.003) (Figure 6b).

In trials where MOM or DHM was maintained as the baseline diet in both arms, HumBMF may result in little to no effect on head circumference gain (3 RCTs, 125 infants, MD −0.00 cm/week (−0.09, 0.10), *p* = 0.94, *I*^2^ = 0%). However, in dual-intervention trials, this combined intervention (HM and HumBMF) may increase head circumference gain (1 RCT, 145 infants, MD 0.08 cm/week (0.01, 0.15), *p* = 0.03). There was no evidence that the effect of HumBMF differed according to whether HM (either MOM or DHM) was maintained as the baseline diet in both arms, or whether formula was used to supplement insufficient MOM in the control group (interaction *p* = 0.21) (Figure 6c).

#### 3.4.5. Duration of Hospital Stay

Two studies reported duration of hospital stay; in both, the control group received formula to supplement insufficient MOM [29,31]. Both studies reported little to no difference between groups. The results were not pooled, however, because duration of hospital stay was reported as medians with interquartile ranges. Conversion to means and standard deviations was not undertaken as the reported medians and interquartile ranges suggested skewed distributions, and the estimated standard deviations were large, making pooled mean differences difficult to interpret.

### 3.5. Subgroup Analysis

There were insufficient data to assess differential effects by gestational age.

### 3.6. GRADE Assessment

We conducted separate GRADE assessments according to baseline diet group, because the clinical context differed substantially between studies in which MOM or DHM was maintained as the baseline diet in both arms and studies in which the control arm received formula to supplement insufficient MOM. Trials in which DHM replaced formula evaluated a dual feeding strategy rather than the independent effect of fortifier composition. Overall, the certainty of evidence ranged from very low to high. In the group where MOM or DHM was maintained as the baseline diet in both arms, certainty was generally low to moderate.

Evidence for mortality, NEC (including surgical NEC), neurodevelopmental impairment, and feeding intolerance was downgraded for serious or very serious imprecision, reflecting small sample sizes, few events, and wide confidence intervals. NEC outcomes were additionally downgraded for risk of bias where the evidence was substantially contributed by open-label trials without reported blinded outcome assessment. Evidence for ROP, sepsis, and BPD was of moderate certainty. In the group where the control arm received formula to supplement insufficient MOM, certainty ranged from very low to high. Evidence for mortality and feeding intolerance was judged to be very low certainty because of substantial imprecision, with mortality additionally downgraded for inconsistency. Evidence for NEC was of moderate certainty, with no downgrade for risk of bias, while evidence for surgical NEC was of low certainty because of imprecision and inconsistency. Evidence for ROP and sepsis was of moderate certainty, whereas evidence for BPD was of high certainty. Across both groups, imprecision was the most common reason for downgrading the certainty of evidence, reflecting the limited number of studies, small sample sizes, and low event rates for many outcomes. Risk of bias contributed to downgrading only for feeding intolerance, a subjective outcome potentially influenced by lack of blinding (Table 2 and Table 3).

### 3.7. Post Hoc Sensitivity Analysis

Sensitivity analyses using random-effects models produced similar point estimates to the primary fixed-effect analyses for most outcomes (Table 4). Confidence intervals were wider for some outcomes with few contributing trials, particularly NEC and surgical NEC in the combined feeding-strategy comparison.

## 4. Discussion

### 4.1. Summary of Main Results

This systematic review included nine RCTs comparing HumBMF with BovBMF in preterm infants receiving human milk. However, an important distinction emerged between studies. Six trials compared fortifier type while maintaining the same baseline milk diet (MOM and/or DHM) in both groups, allowing estimation of the independent effect of the fortifier. In contrast, three trials simultaneously co-randomised both the fortifier and the type of supplemental milk, with infants receiving DHM in the intervention group and preterm cow milk formula in the control group when MOM was insufficient. Consequently, these studies evaluated a combined dietary strategy rather than the effect of fortifier alone.

When analyses were conducted separately according to these differing clinical contexts, trials comparing fortifier type alone did not provide convincing evidence that HumBMF reduced mortality, NEC, sepsis, ROP or feeding intolerance, or improved growth or neurodevelopment compared with BovBMF; confidence intervals were wide for most outcomes and consistent with both benefit and harm. In contrast, trials evaluating the combined dietary intervention suggested reductions in NEC and improved growth. These apparent benefits cannot be attributed to HumBMF itself because the intervention simultaneously replaced preterm formula with DHM. Furthermore, the apparent reductions in NEC and surgical NEC were not robust to the choice of meta-analysis model, with confidence intervals crossing the null in random-effects sensitivity analyses. This further highlights the uncertainty around the apparent benefit of the combined feeding strategy.

### 4.2. Possible Mechanisms

HumBMF may confer biological advantages if bioactive components naturally present in HM, including immunoglobulins, human milk oligosaccharides (HMOs), lactoferrin, growth factors, and anti-inflammatory mediators, are preserved during processing. These bioactive factors are thought to promote intestinal maturation, strengthen epithelial barrier function, modulate immune responses, and support favourable microbial colonisation, all of which could reduce susceptibility to NEC [34,35]. In particular, specific HMOs such as disialyllacto-N-tetraose (DSLNT) have been associated with a lower risk of NEC in preterm infants, providing further biological rationale for preserving human milk-derived bioactive components [36]. However, pasteurisation alters the structure and biological activity of some proteins, including lactoferrin and immunoglobulins, whereas HMOs are relatively well preserved. Consequently, HumBMF may not maintain the bioavailability of all bioactive components. Moreover, because liquid HumBMF displaces a substantial proportion of feed volume, any advantage in HM-derived bioactive content may be offset by reduced exposure to MOM, which contains these components in their native, unprocessed form.

Despite these biologically plausible mechanisms, our review found little evidence that HumBMF alone reduced NEC or mortality when both groups received the same background human milk diet. In contrast, reductions in NEC were observed only in trials where the intervention simultaneously included DHM while the control group received preterm formula when MOM was insufficient.

One possible explanation is that DHM retains many protective properties of human milk despite some reduction in bioactivity following pasteurisation [37]. Consequently, replacing formula with DHM may reduce intestinal injury irrespective of fortifier type. Conversely, when formula is used to supplement insufficient MOM, increased exposure to bovine components and the absence of human milk bioactive factors may increase intestinal vulnerability, making the combined intervention of DHM plus HumBMF appear more beneficial. However, because these studies altered both the fortifier and the supplemental milk simultaneously, the independent contribution of HumBMF cannot be determined.

These findings contribute to ongoing uncertainties regarding whether HM is intrinsically protective or whether bovine milk components are harmful. For ethical reasons, exposure to MOM cannot be evaluated in RCTs, although observational evidence consistently demonstrates substantial health benefits of HM feeding. Likewise, when DHM or commercial HM products are compared with formula, it is difficult to distinguish whether improved outcomes result from the protective effects of HM bioactive components or from avoiding exposure to bovine proteins and other bovine-derived antigens [4]. Although pasteurisation reduces the activity of some bioactive proteins, DHM retains protective components and has consistently been associated with a lower risk of NEC than formula [37].

Trials in which both groups received the same background HM diet provide the best available evidence to isolate the independent effect of fortifier composition. Their findings suggest that exposure to BovBMF alone is not the principal trigger for NEC, and that the lower NEC rates reported in studies evaluating an exclusive human milk diet are more likely attributable to replacing formula with DHM rather than to the use of HumBMF. The absence of consistent effects on other clinical outcomes may also reflect the high baseline exposure to HM across most included studies, leaving limited opportunity for HumBMF to provide additional benefit.

NEC almost certainly represents a heterogeneous syndrome comprising multiple pathogenic pathways, many of which are not diet-related, which may explain why modifying a single dietary component had limited effects. Unlike mortality, NEC is poorly defined, and there is no consensus on diagnostic criteria, which may introduce bias, especially in non-blinded RCTs [32]. Differences in protein, energy, mineral, and micronutrient composition between fortifiers may also influence growth; however, current evidence does not demonstrate consistent or clinically important differences in growth outcomes attributable to fortifier type alone.

### 4.3. Strengths and Limitations of This Review

The major strength of this review is the recognition and prospective handling of important clinical heterogeneity between studies. Rather than pooling fundamentally different interventions, we prespecified analyses according to whether baseline milk feeding remained constant between groups. This approach allows a more clinically meaningful interpretation of the evidence and avoids attributing effects to fortifier type alone if milk type is also randomised.

Additional strengths include a comprehensive literature search, duplicate screening, outcome-level ROB2 assessment, GRADE, and adherence to contemporary systematic review methodology.

Several limitations should also be acknowledged. First, many outcomes were supported by limited numbers of events and wide confidence intervals, resulting in imprecise effect estimates despite the inclusion of nine randomised trials. Random-effects sensitivity analyses produced similar point estimates for most outcomes, although confidence intervals were wider for some outcomes with few contributing trials, particularly NEC and surgical NEC in the combined feeding-strategy comparison. Certain outcomes such as NEC may also be subject to bias in non-blinded studies [32]. Second, analyses according to background milk feeding were based on prespecified study-level intervention characteristics rather than formal subgroup analyses of participant-level data and should therefore be interpreted cautiously. Third, variation in fortifier composition, nutritional protocols, and outcome definitions may have contributed to clinical heterogeneity. Finally, long-term neurodevelopmental outcomes remain poorly studied.

One important methodological issue highlighted by this review is that existing risk-of-bias tools, including RoB2, appropriately assess the internal validity of randomised trials but do not identify whether co-interventions alter the intervention being evaluated. In several included studies, simultaneous randomisation of fortifier type and supplemental milk meant that the intervention no longer represented the independent effect of HumBMF. This issue primarily affects the directness and interpretability of the evidence rather than its internal validity. By prespecifying separate analyses according to background milk feeding, we sought to minimise this limitation and avoid inappropriate attribution of the effects of an exclusive human milk diet to fortifier type alone. Future evidence syntheses may benefit from explicitly considering intervention complexity or co-interventions alongside standard risk-of-bias assessments when evaluating nutritional interventions.

### 4.4. Overall Completeness and Applicability of Evidence

Notably, the three dual-intervention trials were funded or sponsored by a manufacturer of HumBMF [29,30,31], whereas most fortifier-only trials were investigator-initiated. Although these studies were not judged to be at increased risk of bias using ROB2, the differing intervention designs and funding context should be considered when interpreting the evidence.

The included studies were conducted in high-income neonatal intensive care settings and enrolled the populations most likely to receive fortification, including extremely preterm, very preterm, and very low birth weight infants, supporting applicability to similar healthcare settings. However, feeding strategies, fortifier formulations, and nutritional protocols varied considerably across studies. Differences in the use of DHM or preterm formula when MOM was insufficient, the timing of fortifier introduction, enteral feed advancement, and nutritional composition may all have influenced treatment effects and limited direct comparability between studies. Current neonatal practice increasingly uses DHM when there is MOM shortfall; however, numerous uncertainties exist for the use of fortification, and the absence of benefit for DHM in reducing NEC requiring surgery or mortality requires further exploration. Applicability to resource-limited settings or healthcare systems where DHM is unavailable, or feeding practices differ substantially, is highly uncertain.

Several recent systematic reviews and meta-analyses, including Reyes et al. (2025) [38], Huizing et al. (2025) [39], Galis et al. (2024) [40] and Aananthan et al. (2020) [41], have evaluated HumBMF versus BovBMF in preterm infants. The Aananthan et al. study was conducted before five additional RCTs became available and pooled studies despite substantial differences in background feeding strategies. Reyes et al. combined randomised and observational studies with heterogeneous feeding exposures, limiting causal interpretation of the independent effect of fortifier type. Huizing et al. restricted inclusion to randomised trials but included only three studies, resulting in limited statistical power. Galis et al., who included only two trials and two observational studies, reported lower mortality but no significant reduction in NEC among infants receiving HumBMF; however, important outcomes including growth, feeding tolerance, neurodevelopment, and certainty of evidence were not comprehensively evaluated.

In contrast, the present review includes all currently available registered and peer-reviewed RCTs and distinguishes studies evaluating the independent effect of fortifier composition from those evaluating a combined dietary intervention. By analysing fortifier-only and dual-intervention trials separately, this review avoids attributing the potential benefits of DHM to the fortifier itself and provides a more clinically relevant synthesis of the evidence. Together with outcome-level ROB2 assessment and GRADE, this review provides the most comprehensive and up-to-date evidence to inform clinical practice, policy, and future research.

### 4.5. Certainty (Quality) of the Evidence

The certainty of the evidence ranged from very low to high across outcomes. Overall, certainty was most downgraded because of imprecision, reflecting the small number of studies, limited sample sizes, low event rates, and wide confidence intervals. Evidence for mortality, NEC, feeding intolerance, and neurodevelopment was generally of low or very low certainty, whereas evidence for ROP, sepsis, and BPD was of moderate certainty, with high-certainty evidence only available for BPD in the dual-intervention studies.

Importantly, the certainty ratings should be interpreted alongside the differing interventions evaluated. Although we performed separate GRADE assessments according to background milk feeding, studies evaluating DHM in addition to HumBMF addressed a combined feeding strategy rather than the independent effect of fortifier composition. Consequently, even outcomes supported by moderate- or high-certainty evidence may not directly answer the clinical question of whether HumBMF alone is superior to BovBMF.

## 5. Conclusions

Current evidence does not demonstrate clear or consistent clinical advantages of HumBMF over BovBMF when both are added to the same background HM diet. Apparent reductions in NEC and improvements in growth were observed primarily in studies evaluating a combined dietary intervention in which DHM replaced preterm formula when MOM was insufficient. These findings suggest that the observed benefits are more likely attributable to the combined exclusive human milk feeding strategy than to fortifier composition alone.

These findings should be considered alongside the substantially higher cost of HumBMF, limited availability, the potential displacement of MOM by liquid human milk products, and the ethical and logistical considerations surrounding human milk sourcing, processing, and commercial manufacture. Routine use of HumBMF in settings where BovBMF are already added to a HM diet is not supported by the current evidence.

Future research should prioritise long-term follow-up of existing trial cohorts, cost-effectiveness analyses, and strategies to optimise the provision of MOM and DHM. Future evidence syntheses should distinguish trials evaluating the independent effect of fortifier composition from those evaluating combined exclusive human milk feeding strategies to avoid attributing the benefits of DHM to the fortifier itself.

## Figures and Tables

**Table 1:** Study characteristics of included studies.

Study Authors, Year	Country, Participants	Intervention and Control	Baseline Characteristics (Birthweight, Gestational Age)	Funding Sources
Berrington 2024 [23]	UK April 2022 and December 2022 N = 31 GA < 32 weeks or BW < 1500 g across two sites who had received MOM or DHM or both and had not commenced fortification.	Intervention (n = 16): Fortifier: Freeze-dried powdered HumBMF (Neokare Ltd., Worcestershire, UK) MOM shortfall replacement: DHM (first 21 days only; Principal investigator’s discretion thereafter) Control (n = 15): Fortifier: Powdered BovBMF (Nutriprem [Nutricia Ltd.] or SMA Fortifier [SMA Nutrition] MOM shortfall replacement: DHM (first 21 days only; Principal investigator’s discretion thereafter)	Intervention: BW 955 [633–1288] g; GA 27.6 [24.3–28.8] weeks Control: BW 1040 [810–1480] g; GA 27.3 [26.1–30.4] weeks	Sponsored by Newcastle upon Tyne Hospitals NHS Foundation Trust and funded by Neokare Ltd., which also provided the HumBMF. The trial stopped early after the manufacturer’s withdrawal of powdered HumBMF.
Boehm 1993 [24]	Italy, Germany N = 53 GA < 32 weeks, BW appropriate for GA; tolerance of enteral feeding of at least 150 mL/kg per day; no obvious disease or malformation; no oxygen requirement.	Intervention (n = 17): Fortifier: Human milk protein, 0.6 g/100 mL plus minerals MOM shortfall replacement: DHM Control 1 (n = 18): Fortifier: Eoprot (mixture of bovine proteins, peptides and amino acids), 3 g/100 mL MOM shortfall replacement: DHM Control 2 (n = 18), Fortifier: FM85 (whey protein hydrolysate), 4 g/100 mL MOM shortfall replacement: DHM Control 1 and control 2 have been merged for analysis All formulations were designed to achieve a similar macronutrient composition across groups. Infants received 170–180 mL/kg/day of the allocated feeding regimen for 3 weeks.	Intervention: BW 1295 ± 155 g; GA 30.1 ± 1.2 weeks Control: BW 1345 ± 130 g; GA age 30.3 ± 1.7 weeks	Supported in part by the Consiglio Nazionale delle Ricerche, Progetto Finalizzato, Italy, Target Project on Biotechnology and Bioinstrumentation.
Embleton 2023 [29]	UK March 2018 and September 2019 N = 126 GA < 30 weeks who had only received MOM before 72 h of age	Intervention (n = 63): Fortifier: Liquid HumBMF (P+6, Prolacta Biosciences) (n = 63). MOM shortfall replacement: pasteurised human milk product (RTF 26, Prolacta Biosciences) Control (n = 63): Fortifier: BovBMF (Nutriprem, Nutricia Ltd., or SMA Fortifier, SMA Nutrition UK (Gatwick, UK)) MOM shortfall replacement: Preterm formula	Intervention: BW: 930 [733–1095] g; GA: 27.1 [25.7–28.1] weeks Control: BW: 910 [704–1054] g; GA: 27.0 [26.0–28.1] weeks	Sponsored by Newcastle Hospitals NHS Foundation Trust, Newcastle-upon-Tyne, UK, and funded by Prolacta Biosciences, California, US, which also provided human milk formula and fortifier.
Jensen 2024 [25]	Sweden February 2019 and May 2021 N = 228 Extremely preterm infants with GA between 22 + 0 and 27 + 6 weeks	Intervention (n = 115): Fortifier: Liquid HumBMF (Humavant +6, Prolacta Bioscience) MOM shortfall replacement: DHM Control (n = 113): Fortifier: BovBMF (Brand not specified) MOM shortfall replacement: DHM Infants in both groups were not fed with formula during the intervention period, which ended at postmenstrual week 34 + 0.	Intervention: BW: 793 ± 212 g; GA: 25.6 [24.6–26.7] weeks Control: BW: 787 ± 207 g; GA: 26.0 [24.5–27.1] weeks	Funded by grants from the Swedish Research Council (2020-01111 and 2019-01005), the Research Council for Southeast Sweden, ALF Grants, Prolacta Bioscience, CA, USA.
Mizuno 2026 [30]	Japan 28 October 2021, and 18 March 2023 N = 147 GA < 31 weeks and BW < 1500 g	Intervention (n = 77): Fortifier: Liquid HumBMF (Prolacta Bioscience Inc., Duarte, CA, USA) MOM shortfall replacement: DHM Control (n = 70): Fortifier: Powdered BovBMF (HMS-1/HMS-2, Morinaga Ltd., Tokyo, Japan) MOM shortfall replacement: DHM or preterm infant formula	Intervention: BW: 908 ± 264 g; GA: 27.1 ± 2.6 weeks Control: BW: 948 ± 276 g; GA: 27.6 ± 2.3 weeks	Funded by Prolacta Bioscience Inc. Biostatistical and data analysis programming was provided by Innovative Analytics Inc. and the statistical design of the study was performed by Prolacta Bioscience Inc. Medical writing services were funded by Clinigen K.K. and provided by MIMS Co., Ltd. Prolacta Bioscience Inc. supplied the fortification materials, specifically the HumBMF, and covered the Institutional Review Board (IRB) fees through a Contract Research Organization (CRO).
Nogueira-Pileggi 2022 [26]	Brazil N = 40 BW between 750 and 1500 g	Intervention: Human milk + human milk lyophilisate (LioNeo) (n = 20) Control: Human milk + cows’ milk protein origin (FM 85^®^, Nestlé^®^) (n = 20)	Intervention: BW: 1220 ± 201 g; GA: 30.5 ± 2.7 weeks Control: BW: 1219 ± 205 g; GA: 29.7 ± 1.8 weeks	Supported by the Conselho Nacional de Desenvolvimento Científico e Tecnológico (CNPq), Grant #: 421721/2017-0 to JSC Jr., Bill and Melinda Gates Foundation, Grant # OPP1107597 to JSC Jr.
O’Connor 2018 [27]	Canada August 2014 and November 2015 N = 125 BW < 1250 g	Intervention (n = 64): Fortifier: HumBMF (Prolact+4, Prolact+6 and Prolact+8, Prolacta Bioscience) MOM shortfall replacement: DHM Control (n = 61): Fortifier: Powdered BovBMF (Similac Human Milk Fortifier Powder, Abbott Nutrition, Hoofddorp, The Netherlands) MOM shortfall replacement: DHM	Intervention: BW: 887 ± 208 g; GA: 27.9 ± 2.7 weeks Control: BW: 889 ± 196 g; GA: 27.5 ± 2.3 weeks	Funded by a Programmatic Grant in Food and Health from the Canadian Institutes of Health Research (OptiMoM, FHG 129919). Feeding supplies used as part of routine clinical care (e.g., donor milk, BovBMF) were provided by participating centres. Prolacta Bioscience provided the HumBMF at manufacturing cost.
Polberger 1999 [28]	Sweden August 1994 and October 1995 N = 32 BW between 900 and 1750 g	Intervention: Ultrafiltrated human milk protein (n = 16) Control: Bovine whey protein fortifier Wyeth Nutritionals International (Philadelphia, PA, USA) (n =16) Infants were fed exclusively human milk.	Intervention: BW: 1448 ± 244 g; GA: 30.6 ± 1.8 weeks Control: BW: 1447 ± 231 g; GA: 30.7 ± 2.2 weeks	Supported by Wyeth Nutritionals International, Philadelphia, PA, USA, for providing the whey protein fortifier; and the Nestlé R&D Center in Bjuv, Sweden, for producing the human milk protein preparations.
Sullivan 2010 [31]	USA/Austria N = 207 Preterm infants with BW between 500 and 1250 g	Intervention (n = 138): Fortifier: Liquid HumBMF (Prolact+ H^2^MF, Prolacta Bioscience) MOM shortfall replacement: DHM Control (n = 69): Fortifier: Powdered/liquid BovBMF MOM shortfall replacement: Preterm formula	Intervention: BW: 927 ± 198 g; GA: 27.2 ± 2.3 weeks Control: BW: 922 ± 197 g; GA 27.3 ± 2.0 weeks	Supported by Prolacta Bioscience.

Abbreviations: GA, gestational age; BW, birth weight; MOM, mothers’ own milk; DHM: donor human milk; IQR: interquartile range; SD: standard deviation; HumBMF: human milk-derived fortifiers; BovBMF: bovine proterin-based fortifiers. Data are presented as mean ± SD or median [IQR].

**Table 2:** GRADE assessment for the key outcomes in trials where MOM or DHM was maintained as the baseline diet in both arms.

Outcomes	№ of Participants (Studies) Follow-Up	Certainty of the Evidence (GRADE)	Relative Effect (95% CI)	Anticipated Absolute Effects	
				Risk with Bovine Protein-Based Fortifier	Risk Difference with Human Milk-Derived
Mortality	381 (3 RCTs)	⨁⨁◯◯ Low ^a^	RR 0.60 (0.29 to 1.23)	57 per 1000	23 fewer per 1000 (from 40 fewer to 13 more)
Necrotising enterocolitis (modified Bell’s stage ≥ 2)	393 (3 RCTs)	⨁◯◯◯ Very low ^b,c^	RR 0.83 (0.39 to 1.78)	55 per 1000	9 fewer per 1000 (from 34 fewer to 43 more)
Necrotising enterocolitis required surgery	256 (2 RCTs)	⨁◯◯◯ Very low ^b,d^	RR 1.21 (0.36 to 4.09)	94 per 1000	20 more per 1000 (from 60 fewer to 292 more)
Retinopathy of prematurity	372 (3 RCTs)	⨁⨁⨁◯ Moderate ^e^	RR 0.92 (0.69 to 1.23)	27 per 1000	2 fewer per 1000 (from 8 fewer to 69 more)
Sepsis	421 (4 RCTs)	⨁⨁⨁◯ Moderate ^e^	RR 0.95 (0.66 to 1.38)	202 per 1000	10 fewer per 1000 (from 69 fewer to 77 more)
Bronchopulmonary dysplasia	335 (2 RCTs)	⨁⨁⨁◯ Moderate ^e^	RR 0.86 (0.69 to 1.06)	442 per 1000	62 fewer per 1000 (from 137 fewer to 27 more)
Neurodevelopmental impairment at 18 months (cognitive score < 70)	116 (1 RCT)	⨁⨁◯◯ Low ^f^	RR 0.87 (0.37 to 2.04)	167 per 1000	22 fewer per 1000 (from 105 fewer to 173 more)
Feeding intolerance	353 (2 RCTs)	⨁⨁◯◯ Low ^e,g^	RR 0.99 (0.76 to 1.28)	385 per 1000	4 fewer per 1000 (from 93 fewer to 108 more)
* The risk in the intervention group (and its 95% confidence interval) is based on the assumed risk in the comparison group and the relative effect of the intervention (and its 95% CI). CI: confidence interval; RR: risk ratio					
GRADE Working Group grades of evidence High certainty (⨁⨁⨁⨁): we are very confident that the true effect lies close to that of the estimate of the effect. Moderate certainty (⨁⨁⨁◯): we are moderately confident in the effect estimate: the true effect is likely to be close to the estimate of the effect, but there is a possibility that it is substantially different. Low certainty (⨁⨁◯◯): our confidence in the effect estimate is limited: the true effect may be substantially different from the estimate of the effect. Very low certainty (⨁◯◯◯): we have very little confidence in the effect estimate: the true effect is likely to be substantially different from the estimate of effect.					

Explanations. ^a^ Downgraded two levels for very serious imprecision because the confidence interval was wide and included both substantial benefit and no important effect, and the total sample size/events did not meet the optimal information size. ^b^ Downgraded one level for risk of bias because the evidence was contributed by open-label studies that did not report blinded NEC outcome assessment. ^c^ Downgraded two levels for very serious imprecision because the confidence interval was wide and included both appreciable benefit and appreciable harm, with few events and a small total sample size. ^d^ Downgraded two levels for very serious imprecision because there were very few events and the confidence interval was wide, including both substantial benefit and substantial harm. ^e^ Downgraded one level for imprecision because the confidence interval includes both possible benefit and possible harm. ^f^ Downgraded two levels for very serious imprecision because the confidence interval was wide and included both appreciable benefit and harm. ^g^ Downgraded one level for risk of bias because feeding intolerance is a subjective outcome that may be influenced by lack of blinding, and one contributing study was judged as having some concerns.

**Table 3:** GRADE assessment for the key outcomes in trials where the control arm received formula to supplement insufficient MOM.

Outcomes	№ of Participants (Studies) Follow-Up	Certainty of the Evidence (GRADE)	Relative Effect (95% CI)	Anticipated Absolute Effects	
				Risk with Bovine Protein-Based Fortifier	Risk Difference with Human Milk-Derived
Mortality	480 (3 RCTs)	⨁◯◯◯ Very low ^a,b^	RR 1.01 (0.49 to 2.09)	50 per 1000	1 more per 1000 (from 26 fewer to 55 more)
Necrotising enterocolitis (modified Bell’s stage ≥ 2)	354 (2 RCTs)	⨁⨁⨁◯ Moderate ^c^	RR 0.45 (0.20 to 0.99)	42 per 1000	23 fewer per 1000 (from 33 fewer to 0 fewer)
Necrotising enterocolitis required surgery	333 (2 RCTs)	⨁⨁◯◯ Low ^c,d^	RR 0.32 (0.11 to 0.96)	20 per 1000	14 fewer per 1000 (from 18 fewer to 1 fewer)
Retinopathy of prematurity	480 (3 RCTs)	⨁⨁⨁◯ Moderate ^e^	RR 1.04 (0.76 to 1.43)	259 per 1000	10 more per 1000 (from 62 fewer to 111 more)
Sepsis	480 (3 RCTs)	⨁⨁⨁◯ Moderate ^e^	RR 1.11 (0.69 to 1.80)	151 per 1000	17 more per 1000 (from 47 fewer to 121 more)
Bronchopulmonary dysplasia	480 (3 RCTs)	⨁⨁⨁⨁ High ^f^	RR 1.05 (0.89 to 1.24)	522 per 1000	26 more per 1000 (from 57 fewer to 125 more)
Neurodevelopmental impairment—not measured	-	-	-	-	-
Feeding intolerance	147 (1 RCT)	⨁◯◯◯ Very low ^b,g,h^	RR 1.36 (0.40 to 4.63)	78 per 1000	28 more per 1000 (from 47 fewer to 283 more)
* The risk in the intervention group (and its 95% confidence interval) is based on the assumed risk in the comparison group and the relative effect of the intervention (and its 95% CI). CI: confidence interval; RR: risk ratio “-” None of the included studies report this outcome.					
GRADE Working Group grades of evidence High certainty (⨁⨁⨁⨁): we are very confident that the true effect lies close to that of the estimate of the effect. Moderate certainty (⨁⨁⨁◯): we are moderately confident in the effect estimate: the true effect is likely to be close to the estimate of the effect, but there is a possibility that it is substantially different. Low certainty (⨁⨁◯◯): our confidence in the effect estimate is limited: the true effect may be substantially different from the estimate of the effect. Very low certainty (⨁◯◯◯): we have very little confidence in the effect estimate: the true effect is likely to be substantially different from the estimate of effect.					

Explanations. ^a^ Downgraded one level for inconsistency because moderate heterogeneity was present. ^b^ Downgraded two levels for very serious imprecision because there were very few events and the confidence interval was wide, including both substantial benefit and substantial harm. ^c^ Downgraded one level for imprecision because there were few events and the confidence interval was wide, with the upper limit close to no effect. ^d^ Downgraded one level for inconsistency because substantial heterogeneity was present. ^e^ Downgraded one level for imprecision because the confidence interval includes both possible benefit and possible harm. ^f^ Not downgraded for imprecision because the confidence interval was relatively narrow and excluded large clinically important benefit or harm. ^g^ Downgraded two levels for very serious imprecision because the confidence interval was wide and included both appreciable benefit and harm. ^h^ Downgraded one level for risk of bias because feeding intolerance is a subjective outcome that may be influenced by lack of blinding, and one contributing study was judged as having some concerns.

**Table 4:** Post hoc sensitivity analysis for GRADE outcomes.

Outcome	Comparison	Trial (N)	Fixed-Effect RR (95% CI)	Random-Effects RR (95% CI)
Mortality	Fortifier-only	3 trials	0.60 (0.29, 1.23)	0.60 (0.29, 1.23)
	Dual-intervention	3 trials	1.01 (0.49, 2.09)	0.95 (0.29, 3.18)
NEC ≥ stage 2	Fortifier-only	3 trials	0.83 (0.39, 1.78)	0.84 (0.39, 1.81)
	Dual-intervention	2 trials	0.45 (0.20, 0.99)	0.55 (0.11, 2.73)
Surgical NEC	Fortifier-only	2 trials	1.21 (0.36, 4.09)	1.17 (0.34, 4.09)
	Dual-intervention	2 trials	0.32 (0.11, 0.96)	0.46 (0.03, 5.94)
Sepsis	Fortifier-only	4 trials	0.95 (0.66, 1.38)	0.93 (0.57, 1.50)
	Dual-intervention	2 trials	1.11 (0.69, 1.80)	1.12 (0.70, 1.82)
ROP	Fortifier-only	3 trials	0.92 (0.69, 1.23)	0.62 (0.20, 1.93)
	Dual-intervention	3 trials	1.04 (0.76, 1.43)	1.04 (0.76, 1.42)
BPD	Fortifier-only	2 trials	0.86 (0.69, 1.06)	0.86 (0.70, 1.05)
	Dual-intervention	3 trials	1.05 (0.89, 1.24)	1.08 (0.92, 1.26)
Feeding intolerance	Fortifier-only	2 trials	0.99 (0.76, 1.28)	1.00 (0.77, 1.30)
	Dual-intervention	1 trial	1.36 (0.40, 4.63)	1.36 (0.40, 4.63)

Abbreviations: NEC: necrotising enterocolitis; ROP: retinopathy of prematurity; BDP: bronchopulmonary dysplasia; RR: risk ratio; CI: confidence interval.

## Data Availability

The authors confirm that the data supporting the findings of this study are available within the article and its Supplementary Materials.

## Supplementary Materials

The following supporting information can be downloaded at https://www.mdpi.com/article/10.3390/nu18193288/s1: Supplementary File S1: PRISMA 2020 Checklist, Supplementary File S2: Search strategies, Supplementary File S3: Ongoing studies.
