# Supplementary material for: Human Milk-Derived Versus Bovine Protein-Based Fortifiers for Preterm Infants Fed Human Milk: A Systematic Review and Meta-Analysis
Source: Nutrients. 2026 Oct 7;18(19):3288. doi: 10.3390/nu18193288 (PMC13666894; doi:10.3390/nu18193288)
Supplement: Supplementary file 1 [file nutrients-18-03288-s001.zip › nutrients-4550830-supplementary.pdf]

Supplementary file S1

PRISMA 2020 Checklist

Section and Topic	Item #	Checklist item	Location where item is reported
**TITLE**			
Title	1	Identify the report as a systematic review.	Title
**ABSTRACT**			
Abstract	2	See the PRISMA 2020 for Abstracts checklist.	Abstract
**INTRODUCTION**			
Rationale	3	Describe the rationale for the review in the context of existing knowledge.	Introduction
Objectives	4	Provide an explicit statement of the objective(s) or question(s) the review addresses.	Introduction<br>Last paragraph
**METHODS**			
Eligibility criteria	5	Specify the inclusion and exclusion criteria for the review and how studies were grouped for the syntheses.	Methods - Search Strategy and Selection Criteria
Information sources	6	Specify all databases, registers, websites, organisations, reference lists and other sources searched or consulted to identify studies. Specify the date when each source was last searched or consulted.	Methods - Search Strategy and Selection Criteria
Search strategy	7	Present the full search strategies for all databases, registers and websites, including any filters and limits used.	Supplementary file 2
Selection process	8	Specify the methods used to decide whether a study met the inclusion criteria of the review, including how many reviewers screened each record and each report retrieved, whether they worked independently, and if applicable, details of automation tools used in the process.	Methods - Data Collection and Analysis
Data collection process	9	Specify the methods used to collect data from reports, including how many reviewers collected data from each report, whether they worked independently, any processes for obtaining or confirming data from study investigators, and if applicable, details of automation tools used in the process.	Methods - Data Collection and Analysis
Data items	10a	List and define all outcomes for which data were sought. Specify whether all results that were compatible with each outcome domain in each study were sought (e.g. for all measures, time points, analyses), and if not, the methods used to decide which results to collect.	Methods - Search Strategy and Selection Criteria
	10b	List and define all other variables for which data were sought (e.g. participant and intervention characteristics, funding sources). Describe any assumptions made about any missing or unclear information.	Methods - Search Strategy and Selection Criteria

Section and Topic	Item #	Checklist item	Location where item is reported
Study risk of bias assessment	11	Specify the methods used to assess risk of bias in the included studies, including details of the tool(s) used, how many reviewers assessed each study and whether they worked independently, and if applicable, details of automation tools used in the process.	Methods - Data Collection and Analysis
Effect measures	12	Specify for each outcome the effect measure(s) (e.g. risk ratio, mean difference) used in the synthesis or presentation of results.	Methods - Data synthesis
Synthesis methods	13a	Describe the processes used to decide which studies were eligible for each synthesis (e.g. tabulating the study intervention characteristics and comparing against the planned groups for each synthesis (item #5)).	Methods - Data synthesis
	13b	Describe any methods required to prepare the data for presentation or synthesis, such as handling of missing summary statistics, or data conversions.	Methods - Data synthesis
	13c	Describe any methods used to tabulate or visually display results of individual studies and syntheses.	Methods - Data synthesis
	13d	Describe any methods used to synthesize results and provide a rationale for the choice(s). If meta-analysis was performed, describe the model(s), method(s) to identify the presence and extent of statistical heterogeneity, and software package(s) used.	Methods - Data synthesis
	13e	Describe any methods used to explore possible causes of heterogeneity among study results (e.g. subgroup analysis, meta-regression).	Methods - Data synthesis
	13f	Describe any sensitivity analyses conducted to assess robustness of the synthesized results.	Methods - Data synthesis
Reporting bias assessment	14	Describe any methods used to assess risk of bias due to missing results in a synthesis (arising from reporting biases).	Methods - Data Collection and Analysis
Certainty assessment	15	Describe any methods used to assess certainty (or confidence) in the body of evidence for an outcome.	Methods - Certainty of Evidence
**RESULTS**			
Study selection	16a	Describe the results of the search and selection process, from the number of records identified in the search to the number of studies included in the review, ideally using a flow diagram.	Results - Characteristics of included studies, Figure 1 and Table 1.
	16b	Cite studies that might appear to meet the inclusion criteria, but which were excluded, and explain why they were excluded.	Results - Characteristics of included studies, Figure 1 and Table 1.
Study characteristics	17	Cite each included study and present its characteristics.	Results - Characteristics of included studies, and Table 1.
Risk of bias in studies	18	Present assessments of risk of bias for each included study.	Results - Risk of bias and Figure 2

Section and Topic	Item #	Checklist item	Location where item is reported
Results of individual studies	19	For all outcomes, present, for each study: (a) summary statistics for each group (where appropriate) and (b) an effect estimate and its precision (e.g. confidence/credible interval), ideally using structured tables or plots.	Results and Figure 3-6
Results of syntheses	20a	For each synthesis, briefly summarise the characteristics and risk of bias among contributing studies.	Results and Figure 2-6
	20b	Present results of all statistical syntheses conducted. If meta-analysis was done, present for each the summary estimate and its precision (e.g. confidence/credible interval) and measures of statistical heterogeneity. If comparing groups, describe the direction of the effect.	Results and Figure 2-6
	20c	Present results of all investigations of possible causes of heterogeneity among study results.	NA
	20d	Present results of all sensitivity analyses conducted to assess the robustness of the synthesized results.	Results and Post-hoc sensitive analysis, table 4
Reporting biases	21	Present assessments of risk of bias due to missing results (arising from reporting biases) for each synthesis assessed.	Results -<br>Risk of bias and Figure 2
Certainty of evidence	22	Present assessments of certainty (or confidence) in the body of evidence for each outcome assessed.	Results -<br>GRADE assessment
**DISCUSSION**			
Discussion	23a	Provide a general interpretation of the results in the context of other evidence.	Discussion-<br>Summary of main results
	23b	Discuss any limitations of the evidence included in the review.	Discussion-<br>Overall completeness and applicability of evidence
	23c	Discuss any limitations of the review processes used.	Discussion-<br>Strengths and limitations of this review
	23d	Discuss implications of the results for practice, policy, and future research.	Discussion-<br>Conclusion
**OTHER INFORMATION**			
Registration and protocol	24a	Provide registration information for the review, including register name and registration number, or state that the review was not registered.	Methods – first paragraph
	24b	Indicate where the review protocol can be accessed, or state that a protocol was not prepared.	Methods - first paragraph protocol within PROSEPRO
	24c	Describe and explain any amendments to information provided at registration or in the protocol.	Methods – Data

Section and Topic	Item #	Checklist item	Location where item is reported
			synthesis
Support	25	Describe sources of financial or non-financial support for the review, and the role of the funders or sponsors in the review.	Funding Sources
Competing interests	26	Declare any competing interests of review authors.	Conflicts of interest
Availability of data, code and other materials	27	Report which of the following are publicly available and where they can be found: template data collection forms; data extracted from included studies; data used for all analyses; analytic code; any other materials used in the review.	Data Availability Statement

From: Page MJ, McKenzie JE, Bossuyt PM, Boutron I, Hoffmann TC, Mulrow CD, et al. The PRISMA 2020 statement: an updated guideline for reporting systematic reviews. BMJ 2021;372:n71. doi: 10.1136/bmj.n71. This work is licensed under CC BY 4.0. To view a copy of this license, visit https://creativecommons.org/licenses/by/4.0/

Supplementary file S2

Ovid MEDLINE

1.	exp Infant, Newborn/ or Infant Care/ or Infant, Extremely Premature/ or Infant, Premature/ or Gestational Age/ or Intensive Care Units, Neonatal/
2.	(babe or babes or baby* or babies or gestational age? or infant? or infantile or infancy or low birth weight or low birthweight or neonat* or neo-nat* or newborn* or new born? or newly born or premature or pre-mature or pre-matures or prematures or prematurity or pre-maturity or preterm or preterms or pre term? or preemie or preemies or premies or premie or VLBW or VLBWI or VLBW-I or VLBWs or LBW or LBWI or LBWs or ELBW or ELBWI or ELBWs or NICU or NICUs).ti,ab,kw,kf.
3.	1 or 2
4.	randomized controlled trial.pt.
5.	controlled clinical trial.pt.
6.	placebo.ti,ab.
7.	randomized.ti,ab.
8.	randomly.ti,ab.
9.	trial.ti,ab.
10.	groups.ti,ab.
11.	4 or 5 or 6 or 7 or 8 or 9 or 10
12.	enteral nutrition/
13.	exp Food, Fortified/
14.	exp Dietary Supplements/
15.	(fortif* adj4 ((human or breast or expressed or bovine or cow) adj2 milk*)).mp.
16.	(fortif* adj4 ((mother* or maternal or donor* or bovine or cow) adj2 milk*)).mp.
17.	(supplement* adj4 ((human or breast or expressed or bovine or cow) adj2 milk*)).mp.
18.	(supplement* adj4 ((mother* or maternal or donor* or bovine or cow) adj2 milk*)).mp.
19.	(enrich* adj4 ((human or breast or expressed or bovine or cow) adj2 milk*)).mp.
20.	((fortif* or supplement* or enrich*) adj4 DHM).mp.
21.	((fortif* or supplement* or enrich*) adj4 HM).mp.
22.	((fortif* or supplement* or enrich*) adj4 breastmilk*).mp.
23.	exp Milk, Human/
24.	12 or 13 or 14 or 15 or 16 or 17 or 18 or 19 or 20 or 21 or 22
25.	3 and 11 and 23 and 24

EMBASE

1.	exp "Infant, Newborn"/ or "Infant Care"/ or "Infant, Extremely Premature"/ or "Infant, Premature"/ or "Gestational Age"/ or "Intensive Care Units, Neonatal"/
2.	(babe or babes or baby* or babies or "gestational age?" or infant? or infantile or infancy or "low birth weight" or "low birthweight" or neonat* or neo-nat* or newborn* or "new born?" or "newly born" or premature or pre-mature or pre-matures or prematures or prematurity or pre-maturity or preterm or preterms or "pre term?" or preemie or preemies or premies or premie or VLBW or VLBWI or VLBW-I or VLBWs or LBW or LBWI or LBWs or ELBW or ELBWI or ELBWs or NICU or NICUs).tw,kw,kf.
3.	1 or 2
4.	Randomized controlled trial/ or Controlled clinical study/
5.	random$.ti,ab,kw.
6.	Randomization/
7.	placebo.ti,ab,kw.
8.	((double or single or doubly or singly) adj (blind or blinded or blindly)).ti,ab,kw.
9.	(controlled adj7 (study or design or trial)).ti,ab,kw.
10.	parallel group$1.ti,ab.
11.	double blind procedure/
12.	(crossover or cross over).ti,ab.
13.	((assign$ or match or matched or allocation) adj5 (alternate or group$1 or intervention$1 or patient$1 or subject$1 or participant$1)).ti,ab.
14.	(open adj label).ti,ab.
15.	(quasirandom* or quasi-random* or randomi* or randomly).ti,ab,kw,kf.

16.	(control* adj2 (group? or random*)).ti,ab,kw,kf.
17.	4 or 5 or 6 or 7 or 8 or 9 or 10 or 11 or 12 or 13 or 14 or 15 or 16
18.	"enteral nutrition"/
19.	exp "Food, Fortified"/
20.	exp "Dietary Supplements"/
21.	(fortif* adj4 ((human or breast or expressed or bovine or cow) adj2 milk*)).mp.
22.	(fortif* adj4 ((mother* or maternal or donor* or bovine or cow) adj2 milk*)).mp.
23.	(supplement* adj4 ((human or breast or expressed or bovine or cow) adj2 milk*)).mp.
24.	(supplement* adj4 ((mother* or maternal or donor* or bovine or cow) adj2 milk*)).mp.
25.	(enrich* adj4 ((human or breast or expressed or bovine or cow) adj2 milk*)).mp.
26.	((fortif* or supplement* or enrich*) adj4 DHM).mp.
27.	((fortif* or supplement* or enrich*) adj4 HM).mp.
28.	((fortif* or supplement* or enrich*) adj4 breastmilk*).mp.
29.	18 or 19 or 20 or 21 or 22 or 23 or 24 or 25 or 26 or 27 or 28
30.	exp "Milk, Human"/
31.	3 and 17 and 29 and 30

CINAHL

TX ((human milk OR breast milk OR breastmilk OR donor milk OR donor human milk OR DHM OR maternal milk OR expressed milk) N4 (fortif* OR enrich* OR modular*)) AND ((MH "Infant, Newborn+" OR MH "Infant, Premature+" OR MH "Infant, Extremely Premature+" OR MH "Neonatal Intensive Care Units+" OR ((neonat* OR newborn* OR "new N1 born*" OR preterm OR "pre N1 term*" OR premature OR infant* OR VLBW OR ELBW OR NICU) TI,AB,KW)))

Cochrane Central

#1 MeSH descriptor: [Infant, Newborn] explode all trees

#2 MeSH descriptor: [Infant, Extremely Premature] explode all trees

#3 MeSH descriptor: [Infant, Premature] explode all trees

#4 MeSH descriptor: [Intensive Care Units, Neonatal] explode all trees

#5 (neonat* or newborn* or (new NEXT born*) or preterm or (pre NEXT term*) or premature or infant* or VLBW or ELBW or NICU):ti,ab,kw

#6 ((human milk or breast milk or breastmilk or donor milk or DHM or maternal milk or expressed milk) NEAR/4 (fortif* or enrich* or modular*)):ti,ab,kw

#7 #1 OR #2 OR #3 OR #4 OR #5

#8 #6 AND #7

Supplementary file S3

Ongoing studies

1. Effect of human milk-derived fortifiers on weight gain in preterm infants <1250 g: a randomized controlled trial https://cdek.pharmacy.purdue.edu/trial/NCT05228847/#intervention-46172311

2. CTRI/2022/01/039230 2022 Randomized trial of Human milk derived fortifiers versus bovine milk derived fortifiers in comparing the growth parameters in preterm infants less than 32 weeks of gestation

3. CTRI/2022/04/042037 2022 To compare the efficacy of fortifiers derived from human milk versus bovine milk derived fortifiers for weight gain in preterm infants

4. CTRI/2023/01/048687 2023 A study to compare efficacy of preterm human milk supplemented with human milk derived supplement vs bovine milk derived supplement for better growth of preterm

5. CTRI/2023/01/048975 2023 Breastmilk fortification with human milk derived human milk fortifier-RCT
